# Supplementary figures and images for: Insights on early mutational events in SARS-CoV-2 virus reveal founder effects across geographical regions
Source: PeerJ. 2020 May 21;8:e9255. doi: 10.7717/peerj.9255 (PMC7246029; doi:10.7717/peerj.9255)

Tree scale: 0.001

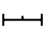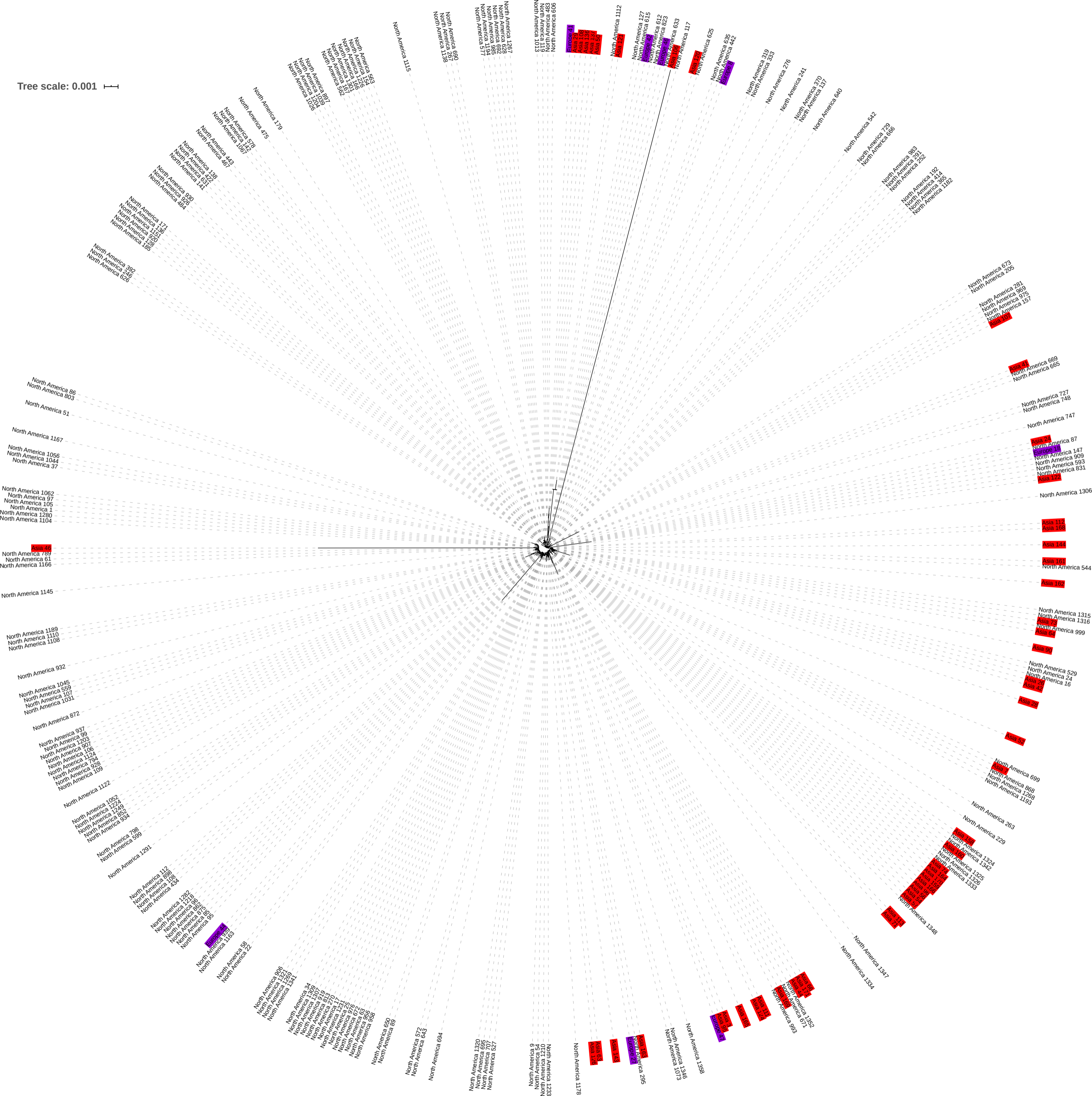

Supplement: Supplemental Information 1 — A phylogenetic tree was constructed by collecting 190, 50 and 1359 sequences from Asia, Europe and North America, respectively. Sequences were aligned by using MAFFT multiple sequence alignment program and an approximately-maximum-likelihood phylogenetic tree was inferred from these alignments with Figtree software. Visualization and editing of the phylogenetic tree were perfomed by using Interactive Tree of Life server (iTOL), collapsing all clades whose average branch length distance was below 0.0002. Asian sequences were highlighted in red, European sequences were highlighted in purple and North American sequences were not highlighted. The tree scale was shown upper. [file peerj-08-9255-s001.pdf]
